# *In-planta* expression of insecticidal proteins provides protection against lepidopteran insects

**DOI:** 10.1038/s41598-019-41833-7

**Published:** 2019-05-01

**Authors:** Imran Rauf, Shaista Javaid, Rubab Zahra Naqvi, Tanveer Mustafa, Imran Amin, Zahid Mukhtar, Georg Jander, Shahid Mansoor

**Affiliations:** 1 Agricultural Biotechnology Division, National Institute for Biotechnology and Genetic Engineering (NIBGE), Jhang Road, Faisalabad, Punjab Pakistan; 2 Pakistan Institute of Engineering & Applied Sciences (PIEAS), Nilore, Islamabad Pakistan; 3 Boyce Thompson Institute, Ithaca, NY 14853 USA; 4 Institute of Molecular Biology and Biotechnology, University of Lahore Main Campus, Defense Road, Lahore, Pakistan

**Keywords:** Plant sciences, Molecular engineering in plants

## Abstract

The development of advanced biotechnological control strategies opens a new era of environment friendly pest management. The current study is part of such an effort, in which we developed a control strategy based on gene pyramiding that confers broad-spectrum resistance against lepidopteran (*Helicoverpa armigera* and *Spodoptera litura*) and hemipteran (*Myzus persicae*, *Phenacoccus solenopsis*, and *Bemisia tabaci*) insect pests. Previously, we reported a double gene construct expressing Hvt and lectin in tobacco (*Nicotiana tabacum*) plants under phloem specific promoters which confers resistance against hemipteran insects. Here we extended our studies by evaluating the advanced generation of these tobacco plants expressing hvt-lectin against lepidopteran insects. Tobacco plants expressing both toxins were tested against *H*. *armigera* and *S*. *litura*. Insect bioassay results showed 100% mortality of *H*. *armigera* within 48–72 hours and 100% mortality of *S*. *litura* within 72–96 hours. Our results suggest that the use of both toxins as a gene pyramiding strategy to control both lepidopteran and hemipterans insects on commercial basis to reduce the use of chemical pesticides.

## Introduction

The losses of crops caused by insect-pests are quite high and it is estimated that more than 10,000 insect species pose serious damage to important food crops^1^. Chemicals like pesticides are now being utilized in much greater quantities against insect pests than in the past. This has led to drastic effects on target organisms (in the form of pesticide resistance and pest resurgence), non-target organisms (*e*.*g*. by killing predators, parasites, parasitoids, and pollinators), the environment, and human health. To overcome these negative impacts of pesticides, scientists have engineered some agricultural crops to produce insecticidal proteins (*Bacillus thuringiensis*, Bt) and have targeted effect on lepidopteran insects without affecting other organisms in the environment^2^. Due to the adaptability of insects, Bt crops continue to be under threat, and many lepidopteran pests have developed resistance to Bt crops^3,4^.

The cotton bollworm (*Helicoverpa armigera*) and armyworm (*Spodoptera litura*) are among the most voracious lepidopteran pests in the world, causing economic damage to many crops. The *Helicoverpa* genus in the Noctuidae includes many important pest species including *H*. *armigera*, *H*. *punctigera*, *H*. *zea* and *H*. *assulta*^5^. *H*. *armigera* is able to cause economic damage to crops estimated to be in couple of billion dollars annually^6,7^. This pest has a diverse host range and attacks more than 180 plants from 45 families including fiber and food crops. After the introduction of Bt crops, *H*. *armigera* damage was reduced to negligible levels. However, as insects have the ability to adapt quickly to new control technology, this pest developed resistance against Bt crops having Cry1Ac toxin. In 2003, *In vitro* studies confirmed the development of resistance in *H*. *armigera* against Cry1Ac, after which numerous cases of resistance against Cry1Ac were reported^3,8,9^. *Spodoptera litura*, another species in the Noctuidae family, is also a cosmopolitan insect herbivore with a wide host range. A specialized migration capability enhances its population spread all over the world^10^. The management of *S*. *litura* has been very difficult, as it quickly develops resistance against chemical control tactics^11–13^. Bt toxins, including Cry2, Cry1FA, Vip3A, Vip3b, were developed as an alternative approach to manage *S*. *litura*, and the potential of these toxins to be used as front line management tactics in transgenic plants has been confirmed^14–16^.

Numerous studies have been done to identify peptides and proteins with insecticidal properties. After the successful evaluation of the synergistic effects of phloem-specific expression of ωatracotoxin (Hvt) from the Australian funnel web spider (*Hadronyche versuta*) and plant lectin (onion leaf lectin) against hemipteran insects, the present study was initiated to evaluate the efficacy of the same constructs against two chewing insects, *H*. *armigera* and *S*. *litura*. Spider venom consists of a diverse mixture of chemical compounds, including peptides and proteins^17^. These compounds have insecticidal properties with a wide range of mechanisms, with significant potential to improve plant resistance against insect pests^18–20^. ω-ACTX-Hv1a (Hv1a), one of the peptides isolated from *H*. *versuta* venom, acts as a calcium channel blocker to target the central nervous system of insects, resulting in abrupt mortality^21^. Lectins, which have been identified in several plant species, are oligosaccharide-binding proteins present in plants that function in defense against pest attack^22^. Plant lectins are abundant in different plant parts, including roots, leaves, bulbs, tubers, and flowers. Different plant lectins have been found to have entomotoxic effect against different insect orders including both sucking and chewing species^19,23–26^. The general mechanism of lectin in insects is to disrupt the epithelial lining of the midgut cells by binding glycoproteins present in the midgut, which leads to different functional and physiological abnormalities like swelling of epithelial cells, microvilli elongation and cell membrane permeability that allows harmful substances into hemolymph, and impaired nutrient absorption^27,28^. Observed entomotoxic activity of lectins includes reduced fecundity, delayed development, mortality, lack of feeding, and abrogated emergence^22^.

In previous study the Hvt-lectin genes were expressed in tobacco plants under phloem specific promoter confer resistance against sucking insects^19^. Here we have extended our previous study and used the same construct to evaluate the toxic effect of both Hvt-lectin, when expressed in combination under phloem specific promoter in tobacco plants against *H*. *armigera* and *S*. *litura*.

## Results

### Plant selection and molecular analysis of transgenic tobacco plants

**Figure 1:**
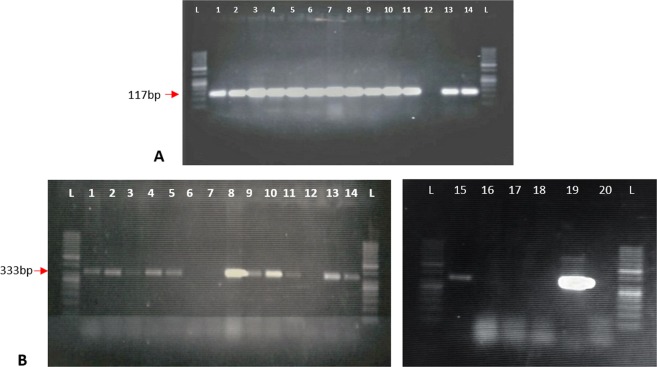
Confirmation of transgenic tobacco. (**A**) Amplification of Hvt from tobacco with gene-specific primers (117 bp); Lane 1–5 (D6 transgenic lines), 6–11 (D15 transgenic lines), 12 (-ve control, non- transgenic line), 13 (+ve control plasmid), L = 50 bp DNA ladder (**B**) Amplification of lectin gene from tobacco with gene specific primers (333 bp); Lane 1–7 (D6 transgenic lines), 8–18 (D15 transgenic lines), 19 (+ve control plasmid), 20 (-ve control, non- transgenic and L = 50 bp DNA ladder.

For the present study, two transgenic lines of tobacco plants expressing the hvt-lectin construct, D6 and D15, were selected. Five biological replicates of D6 and D15 plants in the T_3_ generation were subjected to PCR analysis using primers amplifying an internal segment of the Hvt and lectin genes. All of the selected transgenic plants showed amplification of Hvt and lectin genes with the expected 117 bp and 333 bp product sizes, respectively (Fig. 1A,B).

### Insect bioassays

**Figure 2:**
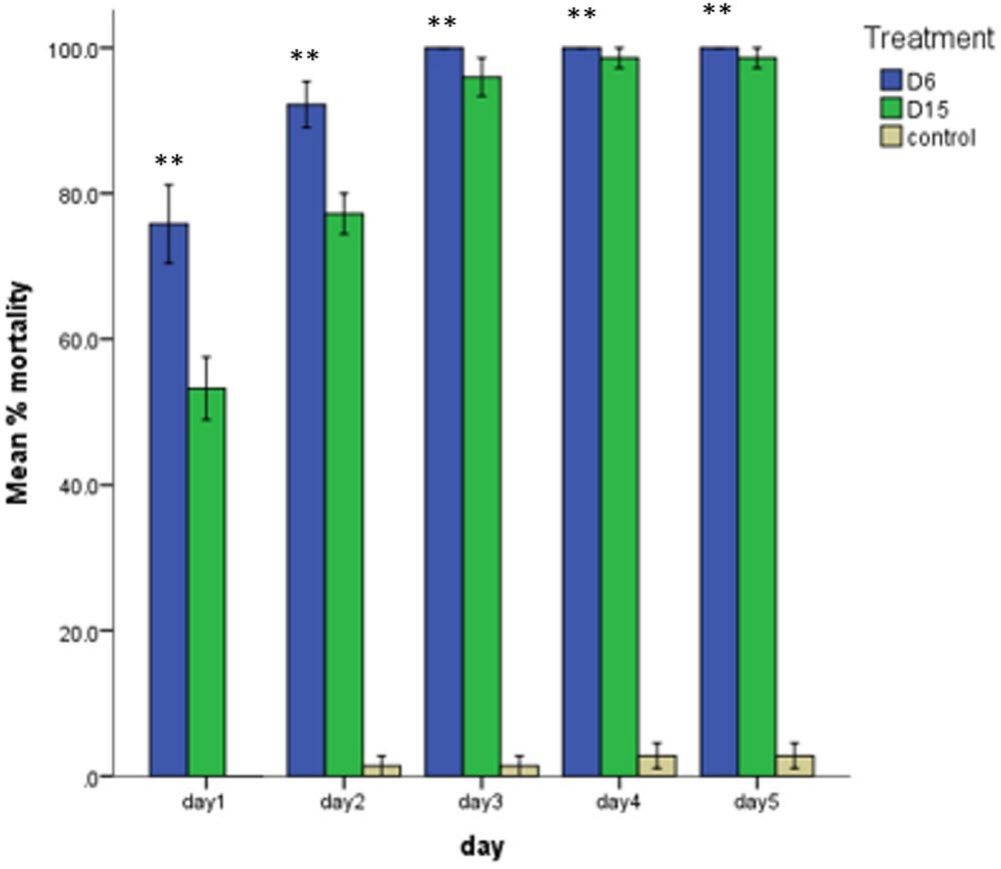
*Helicoverpa armigera* growth on transgenic (D6 & D15) and control tobacco lines. Mean percentage mortality of larvae on lines D6 and D15 expressing Hvt and Lectin toxin proteins. Non-transgenic plants (control) showed the least mortality. Each bar represents the mean +/− s.d. Mean of N = 3, ^**^P < 0.005.

**Figure 3:**
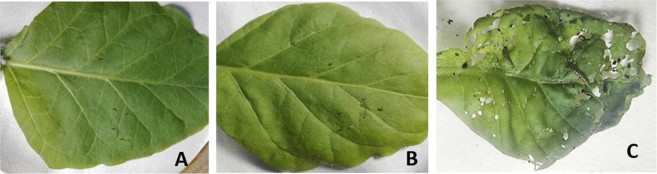
*Helicoverpa armigera* bioassay on transgenic and non-transgenic *Nicotiana tabacum* plants (**A**) Feeding pattern of larvae on D6 transgenic tobacco line (**B**) Feeding pattern of larvae on D15 transgenic tobacco line (**C**) feeding pattern of larvae on non-transgenic tabacum plant.

Bioassays were conducted with transgenic and control plants to evaluate the toxicity of the Hvt and lectin against *H*. *armigera* and *S*. *litura*. Initially, *H*. *armigera* larvae fed voraciously on the leaves, but after 6 hours most of the larvae became sluggish and feeding gradually slowed. After 24 hours, 76% mortality were noted on D6 line and 53% mortality on the D15 line. By the third day, almost 100% of the larvae on D6 leaves were dead. In the case of D15, up to 98% mortality was observed on last two consecutive days (Fig. 2). The effects of Hvt and lectin were also assessed visually, with much less damage being apparent on the D6 and D15 leaves than on control leaves (Fig. 3).

**Figure 4:**
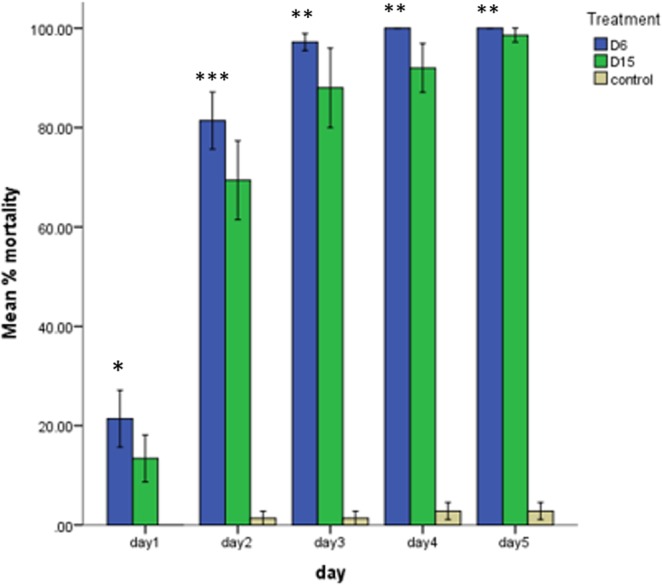
*Spodoptera litura* growth on transgenic (D6 & D15) and control tobacco lines. Mean percentage mortality of larvae on lines D6 and D15 expressing Hvt and Lectin toxin proteins. Non-transgenic plants (control) showed the least mortality. Each bar represents the mean +/− s.d. Mean of N = 3, ^*^P < 0.05, ^**^P < 0.005.

**Figure 5:**
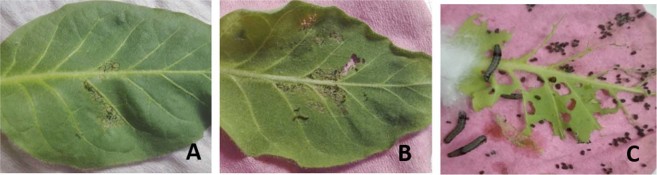
*Spodoptera* litura bioassay on transgenic and non-transgenic *Nicotiana tabacum* plants (**A**) Feeding pattern of larvae on D6 transgenic tobacco line (**B**) Feeding pattern of larvae on D15 transgenic tobacco line (**C**) feeding pattern of larvae on non-transgenic tabacum plant.

The same tobacco plants were used for a detached leaf assay with *S*. *litura*. During the initial period after release, larvae started feeding actively. However, after few hours some larvae looked sluggish and their movement was restricted, whereas others showed some resistance and continued to feed at different intervals. After 24 hours (Day 1) feeding, 21% of the larvae dead on line D6, whereas line D15 showed only 13% mortality. After 96 hours of feeding, 100% mortality was achieved on the D6 line, whereas, on the D15 line there was 92% mortality. The remaining larvae on D15 leaves were allowed to feed for one additional day (120 h), but only 98% mortality was achieved at the end of the day 5 (Fig. 4). As in the case of the *H*. *armigera* experiments, *S*. *litura* larvae consumed much less leaf tissue of the transgenic lines than of control leaves (Fig. 5).

**Figure 6:**
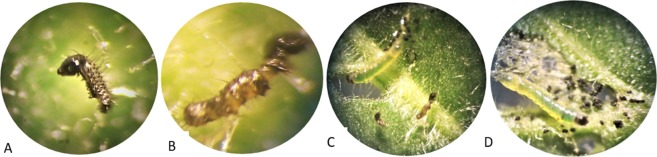
Effects on *S*. *litura* of toxin proteins expressed in transgenic *Nicotiana tabacum* plants. (**A**) Larvae after 48 h feeding (**B**) larvae after 72 h feeding (**C**) comparison of normal and dead larvae (**D**) Normal larvae feeding on a non-transgenic *N*. *tabacum* leaf.

Hvt acts as an antagonist of the insect-specific calcium channel and produce symptoms like lack of coordination, uncontrolled movement, decreased body mass, and browning of the body^19^. The previously described symptoms, including lack of feeding, stunted growth, and changing of color from green to brownish black (Fig. 6) were also observed with *S*. *litura* in the current study.

## Discussion

Keeping in view the world population, the demand for food production is increasing day by day. With likely increases in pest attacks, there is a need for implementation of advanced strategies to counter these pests. Thus, a combination of toxin proteins with different modes of actions is one of the tools being used to improve resistance against chewing insects for the long term. The present study was designed to use the gene pyramiding technique and evaluate the efficacy of two toxin proteins (Hvt and lectin) expressed in tobacco plants under phloem-specific promoters against economically important chewing pests *H*. *armigera* and *S*. *litura*. Our results proved the toxicity of both proteins, and significant mortality was observed in both chewing insects (Figs 2 and 4). Both toxins, in combination, reduced the population of *H*. *armigera* and caused 100% mortality within 48–72 hours, whereas, in the case of *S*. *litura* mortality was confirmed within 72–96 hours. These results are consistent with those of Abbas *et al*.^29^, who reported the same symptoms on *H*. *armigera* and *S*. *litura* larvae within two hours after feeding, when Hvt was applied topically. Similarly, different studies confirmed the significant mortality of *H*. *armigera* and *S*. *litura* in plants expressing Hvt in combination with other insecticidal proteins^20,21,29,30^.

Lectins are another class of toxic proteins used for the management of different insect pests. Mostly, lectins have been used against sucking pests, and their role in controlling lepidopteran insects is still under investigation. Several studies confirmed the effectiveness of different lectins when used with other proteins, as well as the direct effect on physiology in lepidopteran insects^23,31–37^. Our results confirmed the quick toxic effects of both lectin and Hvt against *H*. *armigera* within 24–48 h. The mortality percentage was 76% and 92% after 24 and 48 h respectively, whereas, these results were contrasts with the results from Hvt alone, which caused larval mortality of only 31% or 6% after 24 h and 69% or 46% after 48 h^20,21,38^. As lectin has been reported to have different modes of action: (i) it may disturb the iron metabolism in insect guts specially in Lepidoptera by binding with ferritin^39^, (ii) it may act as a carrier protein to facilitate other proteins reaching the hemolymph^38^, and (iii) it may act on the peritrophic membrane of the gut region, causing deformation in the membrane and disturbing the microvilli, thereby facilitating the movement of other molecules and toxin proteins into hemolymph^40^. As the peritrophic membrane is absent in hemipteran insects and present in lepidopteran insects^40^, it is possible that lectin may facilitate Hvt movement into hemolymph, thereby enhancing the effects on the insect nervous system.

In summary, our results confirmed the toxicity of Hvt and lectin genes against *H*. *armigera* and *S*. *litura* expressed under phloem specific promoter. Combine expression of Hvt-lectin resulted quick mortality of *H*. *armigera* within 24 h and *S*. *litura* in 48 h. Overall 100% mortality of both insects were achieved within 72–96 h. Therefore, expression of these proteins from phloem specific promoters can be used as an alternative approach for long-term management of chewing as well as sucking insects. This is an exciting possibility that needs further demonstration in crop plants under field conditions.

## Methods

### Plant material

T_1_ seeds of D6 & D15 (Hvt: GenBank accession number AJ938032, lectin: GenBank accession number DQ255944, NSP promoter: GenBank accession number AM418568, CP promoter: GenBank accession number AM418565) transgenic *N*. *tabacum* plants, developed by Javaid *et al*.^19^ were used as an initial material for this experiment. T_1_ seeds were germinated on MS0 medium supplemented with 100 mg/L kanamycin in petri dishes to detect at least one copy of the transgene. Transgenic seedlings growing on kanamycin-supplemented medium were maintained further in glass jars and ultimately shifted to the soil. Plants were raised to the T_2_ generation and T_3_ seeds were collected. T_3_ plants of two transgenic events, D6 and D15, were grown in a glasshouse and subjected to PCR amplification for the detection of the transgenes, as well as insect bioassays.

### Molecular analysis of transgenic plants

Genomic DNA of transgenic plants was extracted using the cetyl trimethylammonium bromide (CTAB) method^41^ and then subjected to PCR analysis for the presence of intact toxin cassettes in the plant genome. A reaction mixture of 25 μL was prepared by adding 5 μL of 50 ng plant DNA, 2.5 μL of 10X PCR buffer, 2.5 μl dNTPs (2 mM), 1 μL MgCl_2_ (25 mM), 0.5 μL of each gene specific primer (Table 1), 0.25 μl Taq DNA polymerase (Thermo Fisher Scientific) and deionized water to make up the remaining volume. The reaction was held in a PCR tube and incubated in a thermocycler, programmed as for 5 min preheat at 95 °C and then 35 cycles of having denaturation at 94 °C for 1 min, annealing temperature of 52 °C for 1 min and extension of 1 min at 72 °C, with a final extension temperature of 72 °C for 10 min.

**Table 1:** Specific primers for insecticidal gene amplification.

Primer Name	Primer Sequence
Hvt Forward	5′-CG AAGCTTATGTCACCAACTTGCATACC-3′
Hvt Reverse	5′-ACTCTAGATTAATCGCATCTTTTTACGG-3′
Lectin Forward	5′-CCAAGCTTATGGCCAGGAACCTACTGAC-3′
Lectin Reverse	5′-ACTCTAGATTAGTAGGTCCAGTAGAACC-3′

### Insect rearing

Two chewing insects *H*. *armigera* and *S*. *litura* were used to evaluate the transgenic tobacco plants expressing Hvt-lectin proteins. Different stages of both insects were collected from the field and reared on chickpea flour-based artificial diet^42^ under controlled conditions of 28 ± 2 °C, 75 ± 5% R.H. and 14–10(L:D) h photoperiod. Adults were kept in jars and fed on 10% honey solution. After egg laying and the emergence of larvae, each larva was shifted to a plastic vial containing artificial diet until pupation. Pupae were shifted to adult rearing jars for adult emergence. Multiple generations were obtained to maintain the culture in laboratory conditions. Second-instar larvae of both insect species were used for insect bioassays.

### Insect bioassays

After kanamycin screening and transgene confirmation through PCR, both two transgenic lines D6 and D15 (T_3_ stage) were shifted to a growth room for further growth at 25 ± 2 °C, 60 ± 5% R.H. and 16–8 (L:D) h photoperiod. Five plants (biological replicates) of each line at the 4–5 leaf stage were used for insect bioassays using the detached leaf method. Three fully expanded leaves from each plant/line were detached, washed with distilled water, and placed in a Petri dishes containing moist filter paper. Five second-instar larvae of *H*. *armigera* or *S*. *litura* were carefully shifted onto each leaf. Petri plates were covered with a lid and sealed with parafilm to prevent escape of the larvae. Mortality data and morphological observations were taken on every 24 hours till 120 hours (day 5). Equal numbers of non-transgenic control leaves were also used to compare the mortality data with transgenic plants. Mortality percentage of the larvae were calculated using the following formula**:**

$$\frac{{\rm{Number}}\,{\rm{of}}\,{\rm{larvae}}\,{\rm{found}}\,{\rm{dead}}\,{\rm{on}}\,{\rm{leaf}}}{({\rm{Total}}\,{\rm{number}}\,{\rm{of}}\,{\rm{larvae}}\,{\rm{allowed}}\,{\rm{to}}\,{\rm{feed}}\,{\rm{on}}\,{\rm{the}}\,{\rm{leaf}}-{\rm{number}}\,{\rm{of}}\,{\rm{larvae}}\,{\rm{that}}\,{\rm{absconded}})}\times 100$$

### Statistical analysis

All data were analyzed by using the statistical software SPSS 21. To check the normality of data ShapiroWilk test was used. ANOVA and Kruskal-Wallis test were carried out to determine the significant differences between treatments at P-value < 0.05
